# An evaluation of mental workload with frontal EEG

**DOI:** 10.1371/journal.pone.0174949

**Published:** 2017-04-17

**Authors:** Winnie K. Y. So, Savio W. H. Wong, Joseph N. Mak, Rosa H. M. Chan

**Affiliations:** 1 Department of Electronic Engineering, City University of Hong Kong, Hong Kong, Hong Kong; 2 Centre for Brain and Education and Department of Special Education and Counselling, The Education University of Hong Kong, Hong Kong, Hong Kong; 3 NeuroSky Hong Kong Research Laboratory, Hong Kong, Hong Kong; Kyoto University, JAPAN

## Abstract

Using a wireless single channel EEG device, we investigated the feasibility of using short-term frontal EEG as a means to evaluate the dynamic changes of mental workload. Frontal EEG signals were recorded from twenty healthy subjects performing four cognitive and motor tasks, including arithmetic operation, finger tapping, mental rotation and lexical decision task. Our findings revealed that theta activity is the common EEG feature that increases with difficulty across four tasks. Meanwhile, with a short-time analysis window, the level of mental workload could be classified from EEG features with 65%–75% accuracy across subjects using a SVM model. These findings suggest that frontal EEG could be used for evaluating the dynamic changes of mental workload.

## Introduction

The construct of mental workload can be understood as the level of cognitive engagement which has a direct impact on the effectiveness and quality of a learning process [1]. While an optimal level of mental workload facilitates efficient learning, mental overload could negatively affect task performance and result in more errors [2]. An overloaded individual may even exhibit psychological symptoms, such as frustration, stress and depression [3]. Yet, there lacks a real-time measure of mental workload which can help an individual identify the optimal level of mental workload and hence enhance one’s learning performance.

Conventionally, the level of mental workload is assessed through the verbal or written feedback of an individual. However, the reliability of such self-reported measurements depends on the metacognition skills of the individual [4]. In an educational setting, a continuous assessment of student’s cognitive engagement can be used to determine the pace of teaching and enhance the effectiveness of the learning process. Nevertheless, it is a challenging task for a teacher to evaluate the cognitive engagement of 30–40 students in a typical classroom setting. Although a teacher can evaluate the learning performance of students based on their coursework and examination(i.e. offline assessment), the immediate need of students during the learning process may not be addressed due to the lack of a real-time assessment of mental workload.

To close the feedback loop in the teaching and learning system, researchers have looked into the use of cutting edge technologies for real-time evaluation of learning performance. For instance, wearable and ambient sensors were used to collect the external environmental information, such as location, surrounding temperature, and people in contact, and provide contextual data in supporting reflective learning of employees in a workplace setting [5]. In a study of behavioral engagement, Liu *et al*. reported that the writing performance of participants was benefited from the feedback of a learning analytic system which determines the level of engagement based on the intermediate states of document development and how the document is modified [6]. However, many of these technologies are task-specific or bounded by the task nature and characteristics.

Recent research has looked into the use of physiological responses to quantify individual mental workload. From animal experiment such as using invasive electrode [7], to human experiment using non-invasive device. Kapoor *et al*. used several body sensors, included eye tracking, mouse sensitivity, skin conductance and chair pressure, to estimate the mental workload of an individual with an accuracy of 80% [8]. Studies have also used EEG technologies to determine mental workload based on brain activities. Hogervorst *et al*. used traditional multi-channel EEG setup to examine the mental workload of 2-minutes period and offered a high classification accuracy (>80%) [9]. So *et al*. correlate the EEG signal to muscle EMG single to investigate motor performance [10]. Nevertheless, the setup of traditional EEG with wet electrodes requires at least 30–60 minutes. Such setting bounds the usage of conventional EEG in a controlled environment, like research laboratory.

Recently, a range of mobile EEG systems, which only have a few electrodes channels and transmit the recorded neural signal to a computer wirelessly, have been developed to measure brain activities outside the laboratory setting [11–13]. For example, Wong *et al*. examined the frontal EEG spectra associated with motor acquisition task using a single channel wireless EEG system [14]. Researchers have also used the dry sensor EEG system to develop a neurofeedback training program for children with Attention Deficit Hyperactivity disorder [12]. Furthermore, mobile EEG has been used in developing Brain-Computer Interface (BCI) for entertainment [15, 16]. Nevertheless, the potential of quantifying mental workload with a mobile EEG system has yet to be explored.

In this study, we aim to examine the feasibility in developing a bio-marker of mental workload based on the frontal activities measured by a mobile single channel EEG system. Previous studies have demonstrated that EEG signals, in particular alpha and theta activities, has a close relationship with cognitive performance and mental effort [17]. In a memory study, Raghayachan *et al*. reported that event-related Theta activity increases with memory load and decreases sharply at the end of the task [18]. However, these findings were obtained from conventional multi-channel EEG with wet electrodes. To investigate how frontal EEG signals collected from a dry sensor may vary with changes of mental workload, four cognitive and motor tasks (i.e. arithmetic operation, lexical decision, mental rotation and finger tapping task) with different level of difficulties were used to elicit a dynamic change of mental workload in this study. Based on the findings of traditional EEG studies, we hypothesized that the level of mental workload can be distinguished based on the alpha and theta activities which are collected at the frontal cortex with a single channel dry sensor EEG system. More specifically, we hypothesized that, relative to tasks with low level of difficulty, higher event-related theta activities would be observed in tasks with hgih level of difficulty. We have also identified the key EEG spectral feature associated with mental workload and explored the feasibility to classify different levels of mental workload from EEG features using Supported Vector Machine(SVM).

## Method

### Experimental design

**Fig 1:**
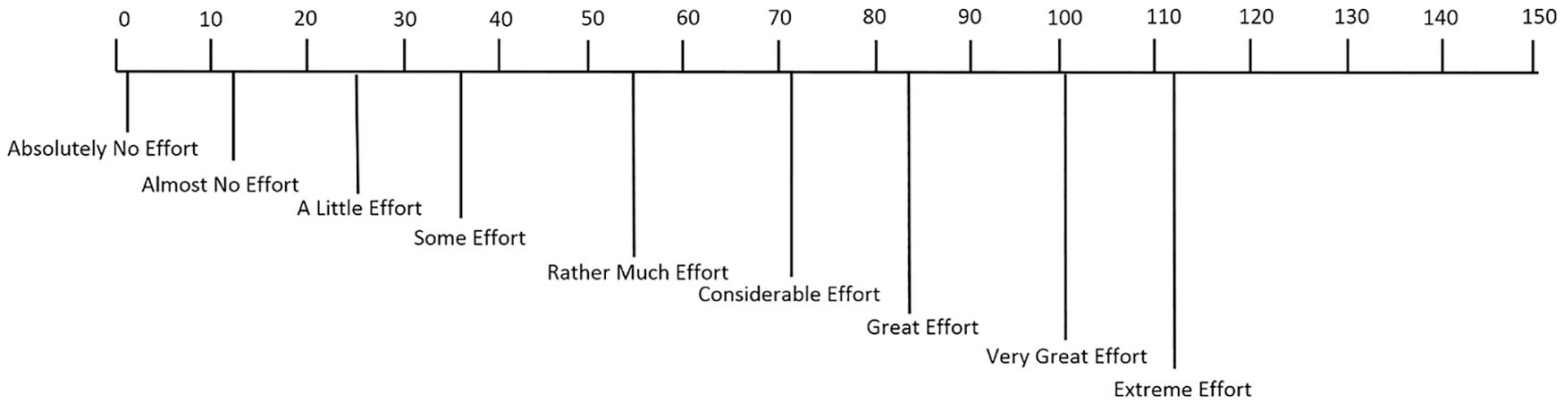
Subjective mental effort questionnaire. SMEQ template for subjective rating used in our experiment [20].

This study consisted of four different cognitive and motor tasks, namely arithmetic operation, finger tapping, mental rotation and lexical decision task which were programmed with Matlab Psychtoolbox [19]. Participants were asked to complete three difficulty levels in the order: low, medium, high for each task. In each difficulty level, there are 25 trials grouped into 5 sessions (i.e. 5 trials per session). In total, each participant had to complete 75 trials of each task. The maximum duration for each trial was 2.5s. After each session, participants gave a subjective mental workload rating on a continuous SMEQ 0–150 range questionnaire [20] see Fig 1. The order of tasks was counterbalanced across participants. The whole experiment lasted for one hour.

#### Arithmetic (Simple calculation task)

In this task, subjects were told to determine the correctness of arithmetic equations showing on the computer screen and responded with button pressing—Left arrow for correct and Right arrow for wrong. Three different difficulty levels were applied in this task—low: single-digit addition, medium: double-digit addition or subtraction with carry set and high: mixed arithmetic operations. Example equations for each difficulty level are shown in Table 1.

**Table 1:** Example questions of three difficulty levels in the arithmetic task.

Low	Answer	Medium	Answer	High	Answer
3 + 4 = 6	F	13 + 18 = 51	F	7 × 3 − 12 = 19	F
4 + 2 = 6	T	61 − 12 = 49	T	63/3 − 32 = −19	T
2 + 7 = 4	F	52 − 67 = −8	F	78 + 11 × 3 = 71	F
3 + 6 = 9	T	−11 + 28 = 17	T	(21 − 13) × 4 = 32	T
5 + 2 = 8	F	−13 − 56 = −60	F	6 × 6 − 79 = −23	F

#### Finger tapping (Visual-motor coordination task)

**Fig 2:**
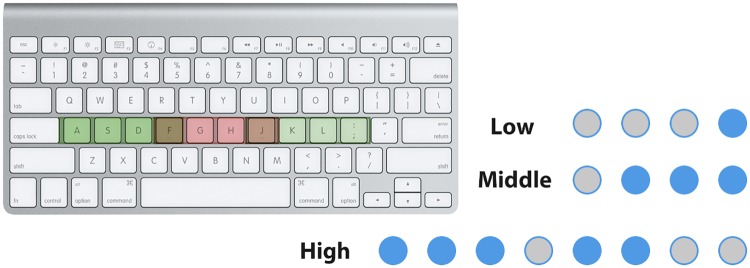
Finger tapping task. Left: finger position with single hand (green) and both hands (red). Right: The blue spots indicate which finger the subject should press.

In this task, subjects were asked to follow the pattern presented on computer screen and perform specific finger tapping pattern on a keyboard. Their wrist and arm maintained stationary, with their fingers other than the thumbs ready on FGHJ buttons and ASDFJKL buttons for single-hand task and two-hand task respectively. Three difficulty levels were established in the task—low: single hand single finger, medium: single hand multiple fingers and high: two hands multiple fingers. For medium and high level trials, subjects were instructed to press all the keys at the same time instead of one-by-one. Fig 2 showed the finger positions and examples of trials.

#### Mental rotation (Visual-spatial task)

**Fig 3:**
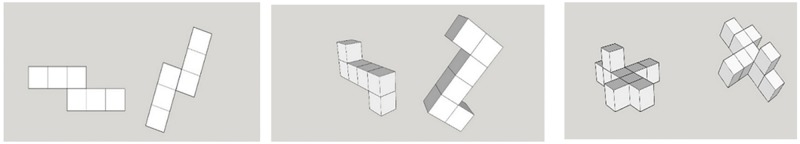
Mental rotation task. 2D, 3D with 6 cubes and 9 cubes in mental rotation task corresponding to low, medium and high level of difficulty.

In the mental rotation task [21], subjects were asked to compared a pair of figures presented on computer screen and determine if they were the same object. The pair of figures could be the same object with different rotation, mirror images or different objects. Easy level contained 6 squares in 2D plane, whereas medium and high level were in 3D space with 6 and 9 cubes respectively. Examples trials were shown in Fig 3.

#### Lexical decision (Linguistic task)

In the lexical decision task [21], subjects were asked to identify whether the stimulus presented was a real English word or a pseudo-word. Different difficulty levels were applied and created by varing the word usage frequency [22], word length and part of speech(Low: concrete noun, Medium/High: noun, adjective, verb). The pseudo-words were generated using the Wuggy software [23]. Examples of each difficulty level are given in Table 2.

**Table 2:** Lexical decision task: Real word and pseudoword used in the three levels of difficulty.

Low		Medium		High	
Real Word	Pseodo Word	Real Word	Pseodo Word	Real Word	Pseodo Word
Butter		narrator		steadiness	
Bread		determined		anomalous	
Hair		dependency		supposition	
Pepper		forecast		expatriate	
	hlnd	carriage	carround	erudition	erumition
	hnajl	moderate	moterate	negligible	nefligible
	eiloul	rehearsal	reheandal	dehydrate	dehyflate
	phnqa	inhabit	inhobit	adjudication	aggudicaiton

A summary of all experimental tasks are in Table 3 and listing out the variations of task content and difficulty level.

**Table 3:** Summary of all the experimental tasks.

Task	Content	Variation	Low	Medium	High	Remark
Arithmetic	Determine whether the arithmetic equation is correct or not	Digit	1	2	2	−
		Arithmetic	+	+ −	+ − */	
		Type	Simple	Simple	Operation	
		Carry Set	No	Yes	Yes	
Finger Tapping	Follow the spot light to press the button on the keyboard	Hand	Single	Single	Both	−
		Fingers	Single	Multi	multi	
Mental Rotation	Judge whether the pair of figures is the same object or not	Dimension	2D	3D	3D	A mirror image of the object is considered as different object
		Number of cube	6	6	9	
Lexical Decision	Determine whether the presented word is a real English word or not	Word Frequency Rank	1–5000	5000–10000	>10000	Word Frequency based on the WORD and PHRASE database Pesudowords are fake words that follow the orthographic and phonological rules of English
		Number of syllable	1–2	3–4	>3	
		word length	4–6	8–10	>9	
		word type	non word	pseudo	pesudo	
		Part of Speech	concrete n	n, v, adj	n, v, adj	

Remark: n-noun v-verb adj-adjustive

### Data collection

Twenty healthy participants (age: 22± 0.71; male/female: 6/14) were recruited in this study. All participants are university students, have normal or corrected-to-normal vision and have no history of neurological or psychological disorder. Participants gave written informed consent to participate by signing the consent form. The experimental procedures were reviewed and approved by the ethics committee at the City University of Hong Kong.

The experiment was conducted in a recording studio room. Participants were told to sit still and relax throughout the experimental. At the beginning of the experiment, 20 seconds of eyes-opened resting state EEG were collected as baseline. Then, participants completed a practice session and became familiarized with the tasks. Data collection began when the participants reached 75% accuracy in 10 consecutive trials or finished 30 trials for each difficulty level. Frontal brain signal was collected at Fp1 channel using a single-channel wireless EEG device (Neurosky MindWave Mobile headset) at 512Hz sampling rate. The ground and referencing was at the lobule. Behavioral data including response time and accuracy were recorded in Matlab. Both the experimental tasks and the EEG recording were controlled with a tablet computer (Surface Pro 3) connected to an external keyboard (Dell L30U).

### Data processing

#### Normalization of subjective rating

To reduce between-subject effect, subjective rating was scaled within subject across tasks into [0, 1] range using Eq 1.

$$\begin{array}{c} X^{\prime }=\frac{X-X_{min}}{X_{max}-X_{min}} \end{array}$$ (1)

**Fig 4:**
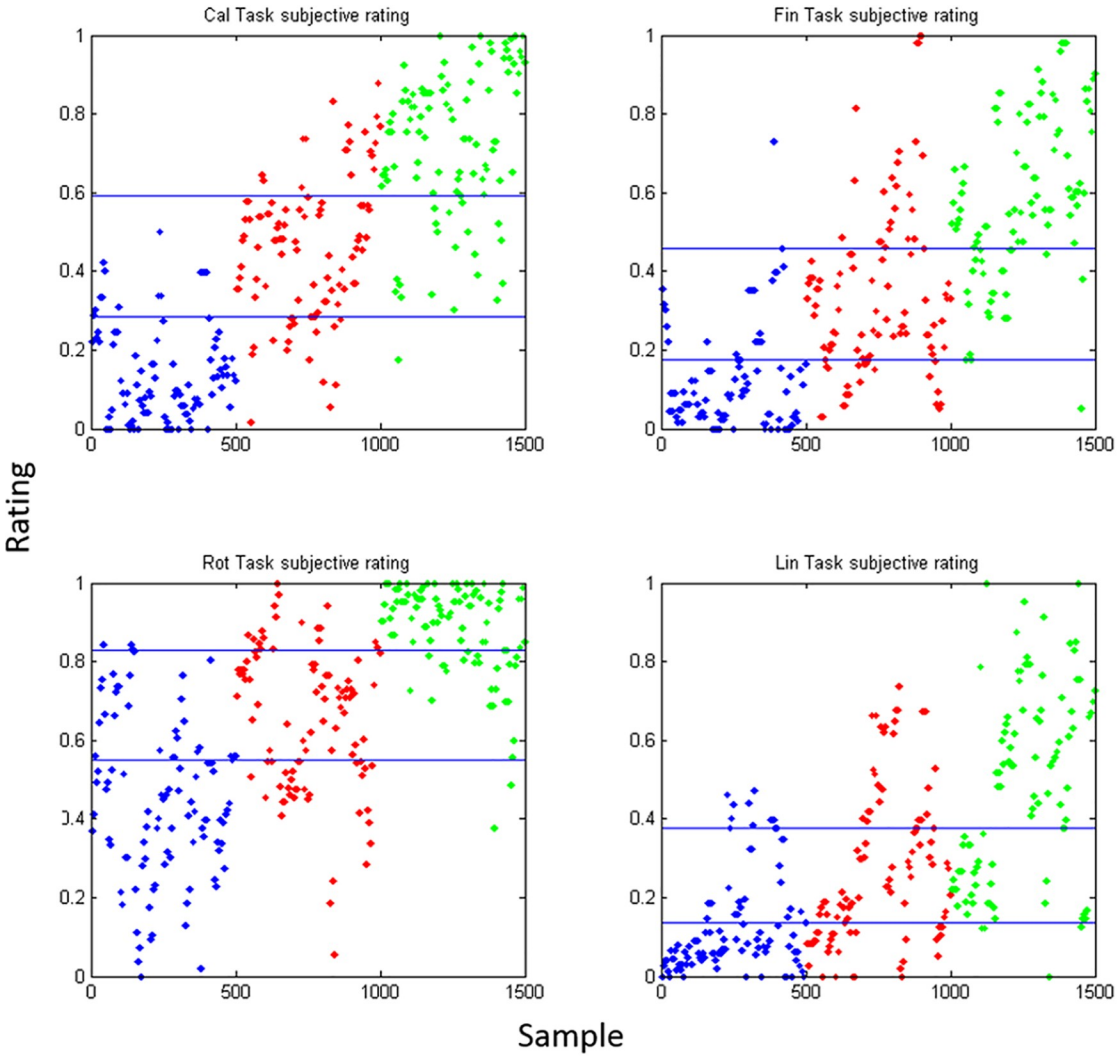
Subjective rating result. The normalized rating of each task. Blue, red, and green indicates low, Medium, and high difficulty levels respectively. The horizontal lines represent the 0.33 and 0.66 quantile respectively.

The normalized between-subjects ratings were then grouped by subjective ratings at 0–33% (low), 34–65% (medium) and 66–100% (high) quantile shown as Fig 4.

#### Pre-processing of EEG signal

Data was first detrened, then bandpass filtered at 0.5 to 45Hz using a FIR filter with a 5^th^ order butterworth window. ICA-based method is commonly used for eye blink and movement artifact removal. Yet ICA requires multi-channel EEG and demand relatively heavier computation. In this study, we employed a wavelet-based filter to the real-time signal channel EEG data to remove eye blink and movement related artifacts [24]. Signal was then segmented into 2.5s epoch according to each trial starting time.

#### Time-frequency analysis

To compute the Time-Frequency matrix (TF Matrix), each trial was segmented with 437.5ms signal window (lower quantile response time of the finger tapping task (low level), 224 data points), with window slide every 31.25ms (16 data points). Welch’s power spectra were calculated over 50% overlapped 2s hamming windows. A trial was rejected if the response time was smaller than 437.5s. Instant Relative power (IRP) is defined as a function of the normalized instantaneous frequency and normalized baseline power in the following form.

$$\begin{array}{c} IRP_{i}=\ln\left(\frac{RP_{i}}{RP_{b}}\right)\;where\;RP_{t}\left(f_{1},f_{2}\right)=\frac{P_{t}\left(f_{1},f_{2}\right)}{P_{t}\left(0.5,45\right)} \end{array}$$ (2)

where (*f*_1_, *f*_2_) is the frequency is range, i is the instant time interval and b is the baseline period.

We computed the EEG frequency band power using two sets of EEG band distributions -traditional EEG frequency band definitions (theta: 4–8Hz, alpha I: 8–11Hz, alpha II: 11–14Hz, beta I: 14–25Hz, beta II: 25–35Hz, gamma I: 35–40Hz, gamma II: 40–44Hz) and individualized frequency band distribution from individual alpha frequency (IAF) [17, 25]. Concerning about individual differences, theta and alpha ranges were defined from the baseline IAF with the following formula [26]. FBIW theta, alpha I and alpha II were defined as (*IAF* − 4) *to* (*IAF* − 2), (*IAF* − 2) *to* (*IAF*) and (*IAF*) *to* (*IAF*+2) respectively. IBIW theta, alpha I and alpha II were defined as (*IAF* × 0.6) *to* (*IAF* × 0.8), (*IAF* × 0.8) *to* (*IAF*) and (*IAF*) *to* (*IAF* × 1.2). We focused on the time course between trial start and key pressing. The average power spectra across the time series were also computed.

#### Visualization of task similarity

Linear and Kernel Discriminate Analysis (LDA, KDA) [27] were implemented to investigate the similarity among the tasks separately. LDA is a supervise dimensionality reduction method which preserves the class discriminatory information. It tried to find the good linear subspace to project the input data and maximize the separation among classes. KDA extend KDA to nonlinear by transforming the space. The kernel operators used is Gaussian kernel.

Both within subject and across subject analyses had been performed. The input features were the EEG power bands and the label information was the task type. We visualized the first two component of disseminate analysis on the x-y plane. The axes are dimensionless after LDA/KDA transformation, which implies no physical meaning.

#### T-Statistics analysis on time-frequency analysis

Here we investigated the oscillatory activities during the task at different difficulty levels. TF matrix from the time frequency analysis was rescaled with respect to completion rate. Each pixel on TF matrix underwent the minimum t statistic for comparison.

After computing the TF analysis, we calculated the t-value [28] for each time-frequency point using 1-sample t-test within each difficulty level for each subject. Next, pairwise comparison between the t-maps of low and high level was performed for the study of largest task differentiability. At last, we worked out the conjunction by finding the minimum t-value between t-maps from pairs of tasks to investigate the common features.

#### Classification of difficulty level

Support vector machine (SVM) [29] was used to classify EEG data at different task difficulty level. Time averaged power of each frequency band was computed from each 2.5s trial, while both subjective and objective task difficulty level were used as class labels. We used a simple 2-class classification first to distinguish the lowest and highest task difficulty levels. Radial basis function kernel (RBF) was used, and repetitions of 10-fold cross validation was.

## Results

### Behavioral result

**Fig 5:**
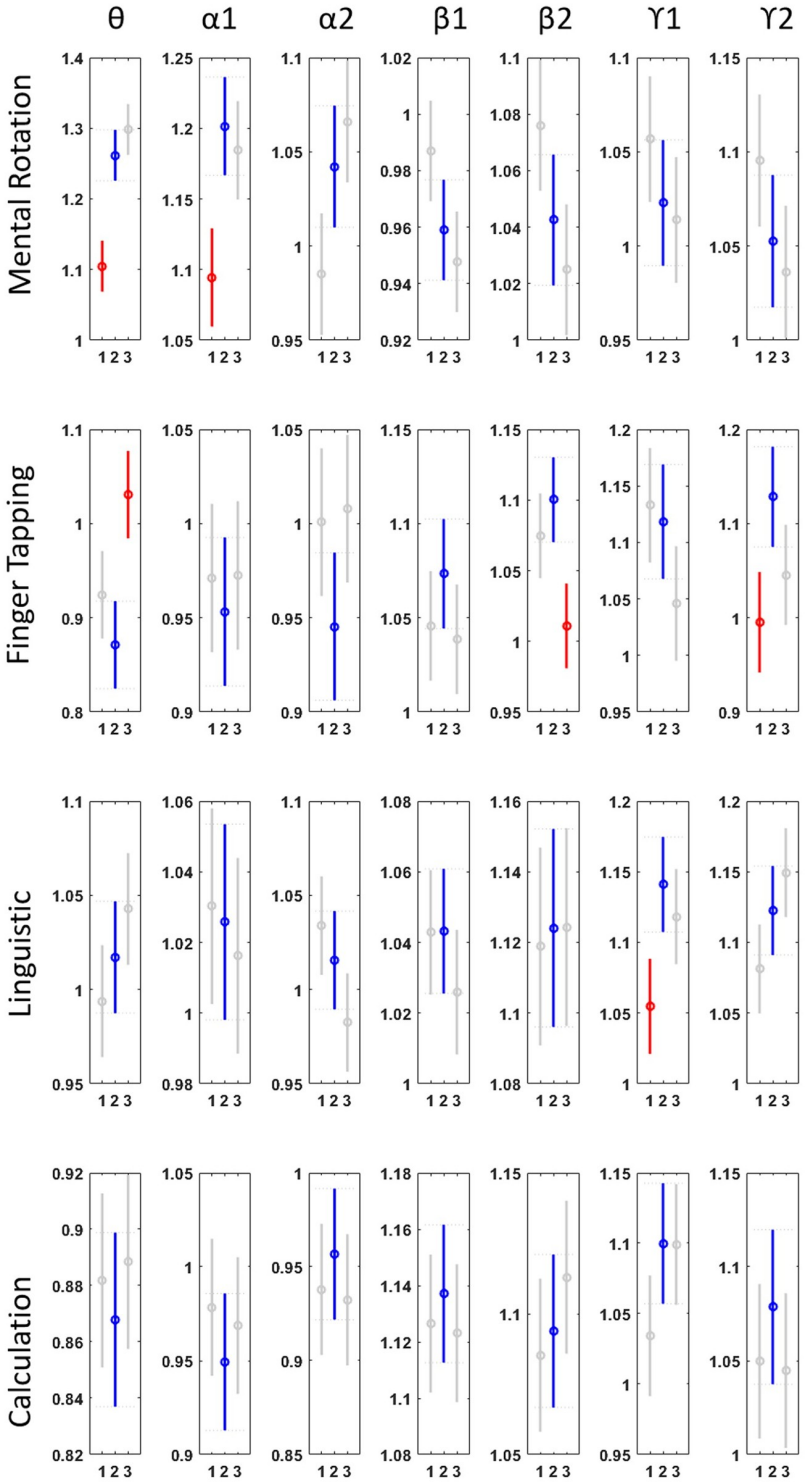
Power of different EEG frequencies in four cognitive task. 1, 2 and 3 are low, medium and high difficulty level respectively. Blue line is indicated the medium level. Red line means it is significantly different from medium level and gray line means not significant.

Repeated measure ANOVA (Table 4) and pairwise analysis (Table 5 and Fig 5)were performed on EEG features and the behavioral performance measures, namely reaction time, missing rate, accuracy and subjective rating within each task. All the behavioral measures exhibited a significant main effect of Task and Difficulty Level (*p* < 0.001). Significant interaction effect between task and difficulty level was observed in the subjective rating (*p* < 0.01), response time, missed rate and accuracy (*p* < 0.001). Post hoc multiple comparisons analysis was conducted to compare each pair of levels. The behavioral data also showed that when the difficulty level increased, the response time and missing rate increased and the accuracy decreased across four tasks. The Spearman correlation between objective level and the subjective rating also showed a significant positive correlation for all four tasks (*p* < 0.05; Table 6). These results implied that the task design successfully created different levels of difficulty for each task. Meanwhile, the difficulty levels between the tasks were not necessarily the same. Because a consistent significant difference in the behavioral measures is observed only between the comparison of low and high difficulty trials (Table 5), therefore the analysis of the EEG data focused on the comparison of the low and high difficulty trials.

**Table 4:** Repeated measure ANOVA of EEG feature and behavioral responses. The within-subjects factors are task (4 levels) and difficulty (3 levels), ***p < 0.001, **p < 0.05 and *p < 0.01.

	Task			Level			Task*Level		
	*F*(3, 17)	*p*	Wilks	*F*(2, 8)	*p*	Wilks	*F*(6, 14)	*p*	Wilks
Subjective Rating	10.069	***	0.36	57.682	***	0.135	2.459	*	0.487
Normalized Rating	19.162	***	0.228	168.718	***	0.051	3.109		0.429
Response Time	313.797	***	0.018	457.459	***	0.19	31.676	***	0.069
Missed	22.754	***	0.199	42.738	***	0.174	15.122	***	0.134
Accuracy	118.634	***	0.046	169.012	***	0.051	18.554	***	0.112
4–8Hz (*θ*)	8.331	**	0.405	9.818	***	0.478	3.66	**	0.389
8–11Hz (*α*_1_)	4.061	***	0.583	5.663	**	0.614	1.756		0.571
11–14Hz (*α*_2_)	5.046	**	0.529	5.639	**	0.615	3.323	**	0.413
14–25Hz (*β*_1_)	1.687		0.771	2.413		0.789	0.561		0.806
25–35Hz (*β*_2_)	2.441	*	0.699	1.433		0.863	0.668		0.777
35–40Hz (*γ*_1_)	1.58		0.782	2.469		0.785	0.319		0.88
40–44Hz ()*γ*_2_)	2.42	*	0.7	2.18		0.805	0.23		0.91

**Table 5:** Statistics of behavioral responses. RT: Response Time(s), Missed: Number of Missed trial/Total Number of Trial, Acc: Accuracy Rate (excluded missed trial), Rating: SMEQ subjective rating scaled to 0–1 range, m: mean value, **p < 0.001 and *p < 0.05.

Task		Low Mean	SD	Medium Mean	SD	High Mean	SD	p	*m*_*low*_ − *m*_*medium*_	*m*_*low*_ − *m*_*high*_	*m*_*medium*_ − *m*_*high*_
Cal	RT	0.96	0.11	1.80	0.21	1.97	0.16	<0.001	−0.85**	−1.02**	−0.17**
	Rating	21.44	0.11	41.70	0.15	58.00	0.19	<0.001	−20.26**	−36.56**	−16.30**
	Missed	0.15	0.02	2.90	0.11	7.35	0.13	<0.001	−2.75**	−7.20**	−4.45**
	Acc	0.95	0.03	0.58	0.03	0.47	0.03	<0.001	0.38**	0.49**	0.11*
Fin	RT	0.58	0.06	0.79	0.13	1.34	0.18	<0.001	−0.21**	−0.76**	−0.55**
	Rating	19.61	0.10	32.02	0.15	48.45	0.18	<0.001	−12.41**	−28.84**	−16.43**
	Missed	0.00	0.00	0.00	0.00	0.20	0.02	<0.05	0.00	−0.20*	−0.20*
	Acc	0.99	0.02	0.92	0.02	0.72	0.02	<0.001	0.07	0.26*	0.20**
Rot	RT	1.66	0.19	1.86	0.17	1.94	0.11	<0.001	−0.20**	−0.29**	−0.09**
	Rating	41.16	0.20	53.63	0.18	67.33	0.17	<0.001	−12.47**	−26.17**	−13.70**
	Missed	1.90	0.09	4.00	0.13	5.40	0.14	<0.001	−2.10**	−3.50**	−1.40
	Acc	0.72	0.03	0.58	0.03	0.44	0.03	<0.001	0.14**	0.28**	0.14**
Lin	RT	0.70	0.08	1.14	0.21	1.40	0.26	<0.001	−0.43**	−0.70**	−0.27**
	Rating	20.08	0.11	29.46	0.20	41.76	0.23	<0.001	−9.38**	−21.68**	−12.30**
	Missed	0.00	0.00	0.30	0.03	0.70	0.04	<0.05	−0.30	−0.70*	−0.40
	Acc	0.98	0.02	0.83	0.02	0.69	0.02	<0.001	0.15**	0.30**	0.15**

**Table 6:** Correlation between subjective and objective rating. * *p* < 0.05 significant difference.

Task	Cal	Fin	Rot	Lin
R	0.813*	0.738*	0.686*	0.586*

### TF analysis: Dynamic change in trials

**Fig 6:**
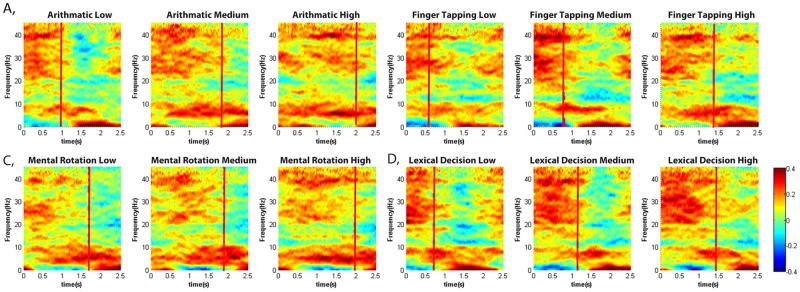
Time frequency analysis of the 2.5s-trial across all the subjects. A: Arithmetic operation, B: Finger tapping, C: Mental rotation, D: Lexical decision. Each Task consists of three difficulty levels.

Fig 6 shows the time-frequency analysis of each task. The baseline has been taken into account using Eq 2. The entire trial from the start point to 2.5s is shown and the vertical line indicates the minimum time response within all the trials. Comparing the low and high difficulty levels, it can be seen there was a relative increase in the frequency components around 20–35Hz when the subject was executing the task. Following completion, frequency components in the 20–30Hz range were suppressed, while the theta range (4–8Hz) increased. We also observed that the response time for the low level is shorter than that for the high level, and we are actually comparing the period during task and after task. This observation represents the change between engagement during task and relaxation after activity.

Medium and high difficulty levels of arithmetic operation and mental rotation require longer response time, we observed the power bursted across the time course in theta and alpha I range. As for the low difficulty level, the shorter the time response, the larger suppression in beta and gamma observed.

### KDA analysis: Identification of task similarity

**Fig 7:**
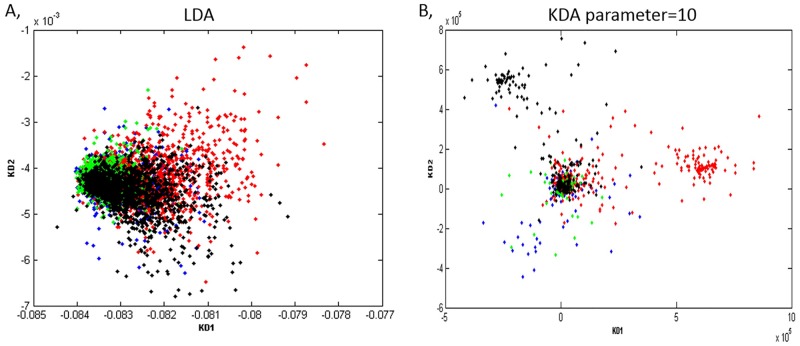
LDA and KDA results. A: LDA without and kernel. B: KDA with all the subjects trials, Gaussian kernel with parameter = 10.

**Fig 8:**
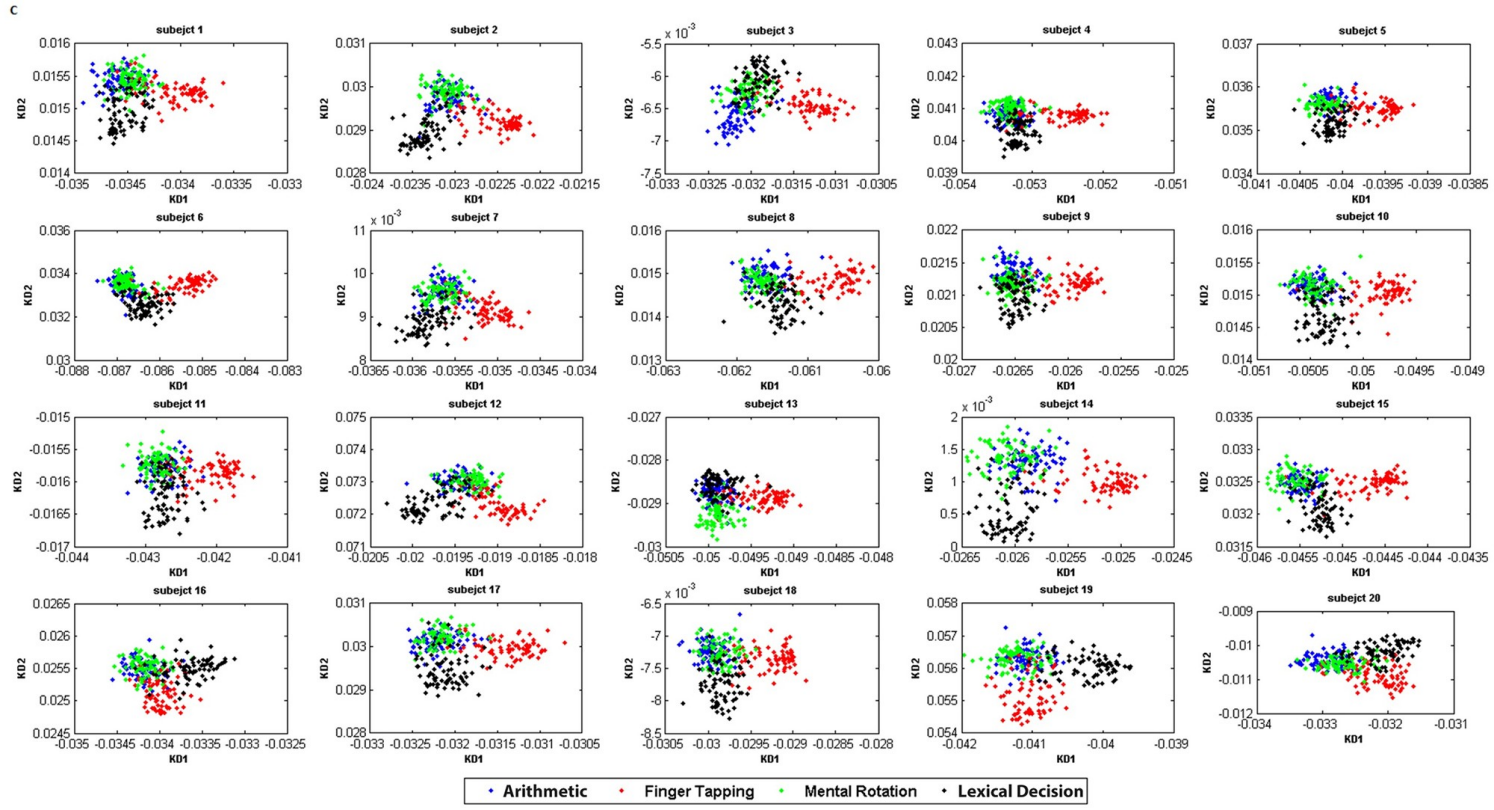
KDA results for each subject. C: KDA within each subject using Gaussian Kernel.

In Figs 7 and 8, the results of KDA transformation with the task class label are presented. Each point presents a trial (Blue: Arithmetic operation, Red: Finger tapping, Green: Mental Rotation, Black: Lexical decision). Different kernel and parameter values were tested. Fig 7A which shows the four tasks overlapping with each other, is transformed by linear discriminate analysis. In Fig 7B, a Gaussian Kernel is used with different parameter values were explored and finally, parameter = 10 showed better results as several clusters are clearly observed. This implies the non-linearity of EEG dynamics, as such that non-linear analysis provides a better approach to characterize the properties.

The KDA transformation for each subject are presented in Fig 8. The distance between pairs of clusters implies how different brain signal changes are among tasks. Shorter distances mean that they were more similar. Generally, arithmetic operation and mental rotation clusters overlapped in most of the subjects, except subjects 3 and 13. On the other hand, finger tapping is more separable from other tasks. From this transformation, we observed the similarity level of the brain signal characteristic among the four tasks. This result demonstrates a possibility to group the cognitive tasks together if we want to build a more general model in the future.

### Changes in band power: Spectral characteristic

**Fig 9:**
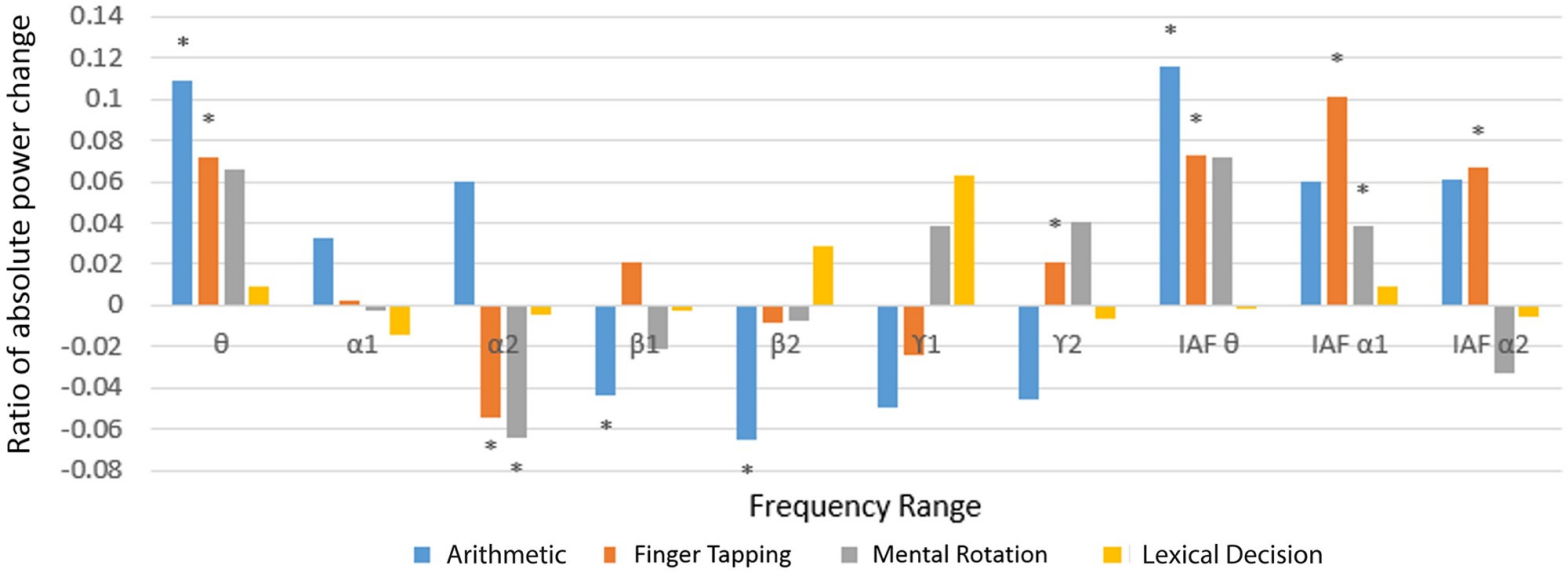
Ratio of absolute power change. Frequency power change between high and low level of difficulty in each task. P-value <0.05.

Fig 9 presents the frequency power change between high and low level of difficulty. The overall changes in arithmetic operation and finger tapping are more significant. An increase in theta power is a common feature among all the tasks. This is consistent with previous findings that theta is related to workload demand [17, 30].

Previous KDA transformation suggested that arithmetic operation and mental rotation are more similar. The frequency power analysis supported this in that they shared the same trend in theta, IAF alpha I and both beta I and II frequency ranges.

Working memory consists of a phonological loop and a visual spatial sketchpad [31]. Among the four tasks, finger tapping and mental rotation rely heavily on the visuospatial loop whereas lexical decision and arithmetic operation involve mainly the phonological loop. Yet, previous research [32] indicated that visual spatial skill also involved in arithmetic operation causing it be more similar with mental rotation. Moreover, both of them require a problem solving process instead of pure long-term memory retrieval or motor coordination.

Fig 9 has showed different patterns of frequency power change which matched with other research groups’ studies. For example, increase of Beta II in lexical decision task referred to the orthographic and semantic difference in the choice of vocabulary [33]. Meanwhile, the gamma increase related to the vocabulary recollection in high difficulty level rather than only familiarity [34]. As for the finger tapping task, the increase in IAF theta, IAF alpha I and II matched our previous study about the motor skill acquisition by using a mirror drawing experiment [35]. These band powers showed that they positively correlated with perceived difficulty level of the task.

### T-statistic of TF analysis

**Fig 10:**
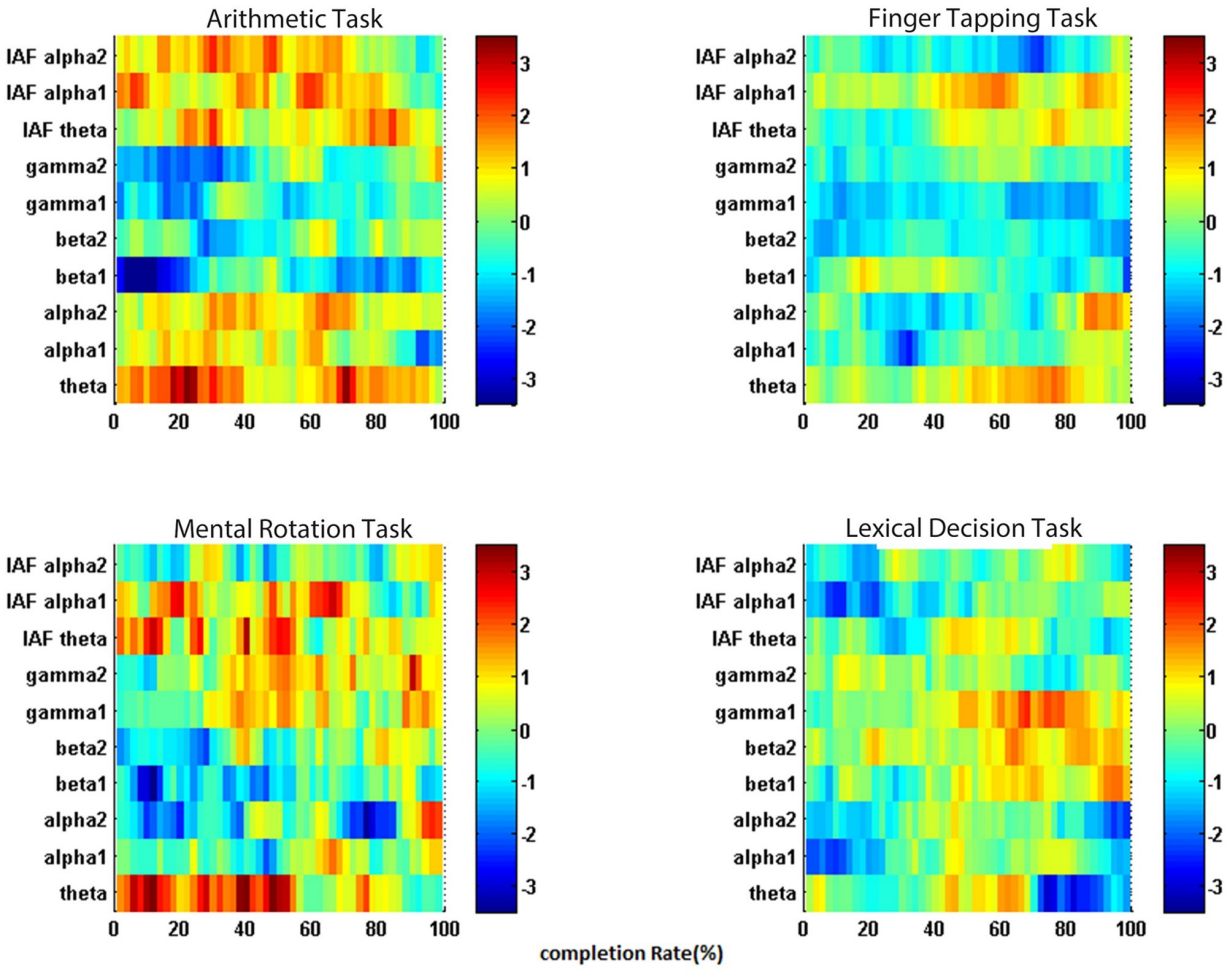
T-statistic map of each task comparing high and low difficulty level.

Time-frequency T-map analysis allowed us to observe the dynamic change in frequency range across the time course. Fig 10 shows the time frequency difference between low and high level after the trial re-sampling. Here, we focus on the change in the common feature, theta activities. Although all four tasks showed an increase in theta, the time of occurrence could differ. Arithmetic operation and mental rotation tasks had theta power increase in over the whole trial whereas it only occurred in the middle of the finger tapping and lexical decision task.

**Fig 11:**
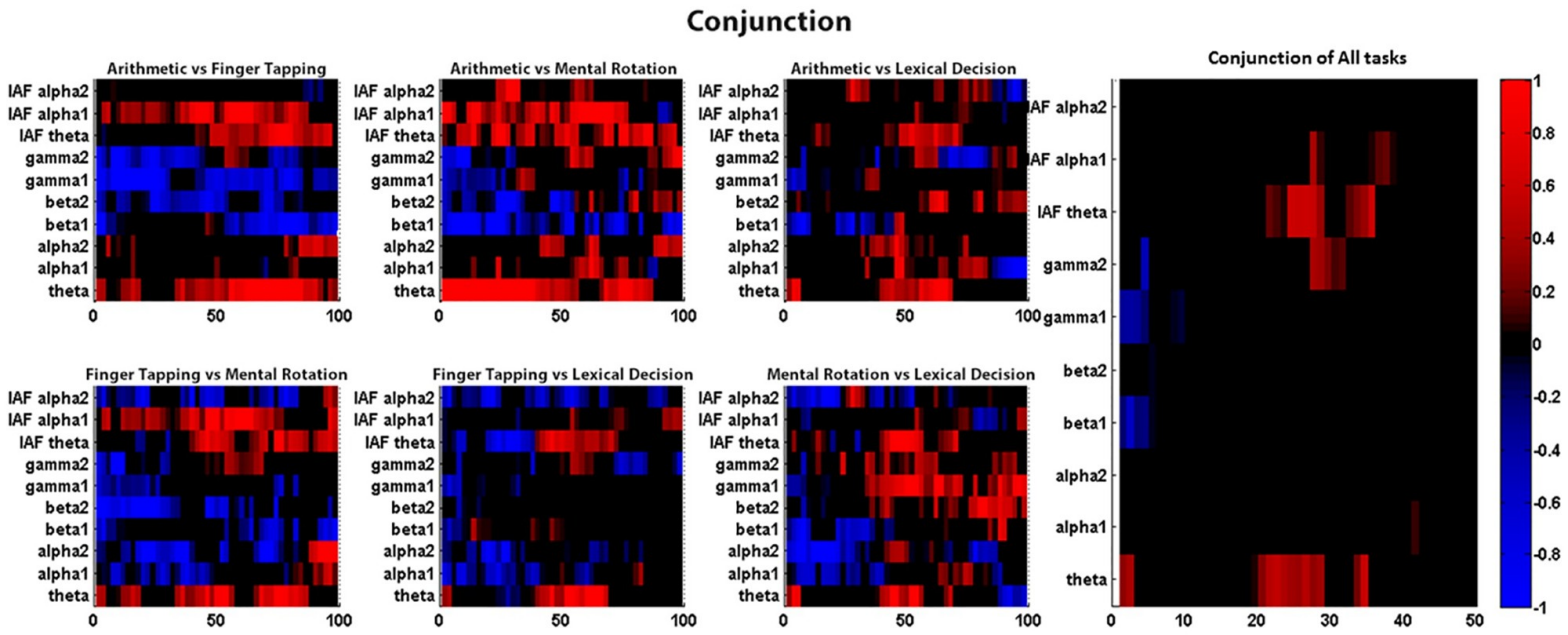
Conjunction of task t-map. (Left) each pair of tasks, (Right) four tasks conjunction.

Fig 11 is the conjunction between two pairs of tasks and showing the minimum T value. Color in red and blue mean the common synchronization and desynchronizaton in two tasks respectively. The blank color means both task were in opposite trend. The rightmost graph is the conjunction of all the tasks and we discovered that theta, beta and the IAF analysis have same trend in synchronization or desynchronization in some time points.

### SVM classification: Real-time analysis model

As a practical application, ability to predict the workload using the frequency power feature by classification technique is necessary. Preliminary result in Fig 5 shows that EEG features from the medium level of difficulty always overlap with either low or high level. To simplify the classification model, we have studied the cases with the lowest and highest difficulties. Table 7 presents the test sample accuracy in 10-fold cross validation during 2-class SVM classification. The samples were taken from across all the subjects and the accuracy reached greater than 70% in Arithmetic operation, finger tapping and lexical decision. Mental rotation had a relative lower accuracy of around 64%, because of the relatively small difficulty gap between the levels as indicated in Fig 4. This test has demonstrated the potential real application of short term prediction of the mental workload using a single channel EEG device.

**Table 7:** Testing sample accuracy of 2-class SVM classification.

	Cal	Fin	Rot	Lin
Objective	75.40%	76.00%	60.40%	74.42%
Subjective	73.91%	73.31%	64.91%	73.03%

## Discussion

This study aims to develop an EEG-based mental workload-detection application by building a generalized model for four different cognitive and motor tasks. Our findings showed that the frontal theta activity is a common feature across these tasks. This result is consistent with previous studies that theta activities increase with the level of mental effort [17, 18]. Meanwhile, the correlation of mental workload level and other frequency bands is task-dependent [3, 36].

With a 2.5s analysis window size, the accuracy of mental workload classification could reach 65%–75%, which is slightly higher than other EEG studies with around 60% accuracy [36, 37]. It might be related to three factors: intra subject variance, task duration and the selection of EEG channel.

First, previous studies have reported that individually adjusted frequency bands are useful for the analysis of event-related potential [26]. The individual differences in alpha peak were evaluated by computing the IAF-defined power value from baseline. Second, unlike previous study which took long measurement and had subject to give an overall rating afterwards, the current experiments was composed of very brief trials and required participants to feedback on the subjective mental workload level immediately after each session. The short-time analysis window indicated the moment when the subjects were engaging in the task. Indeed, as Dai *et al*. suggested that using all channel in the analysis might cause large variance and result to a poorer classification of the task [38]. Active EEG channels should be selected whereas unrelated channels should be discarded in order to improve the accuracy. Our findings illustrated that EEG signals collected from a single-channel dry sensor at Fp1 provide sufficient information in generating a reasonable bio-marker of mental workload.

This study takes the first step in bringing laboratory research technique to real life application with the latest mobile EEG technology. Our findings indicated that event-related frontal EEG theta frequency band power is a common feature of mental workload across different cognitive and motor tasks. The advantage of this model is the ability to detect short term mental workload in real time. Due to the limited sample size of the current study, counterbalance was performed on the order of the four different tasks but the difficulty level. Although the current design resembles to the real-world practice that we usually complete the relatively easy task before heading to the difficult one, we could not rule out the possible practice effect across the three difficulty levels within each condition. The practice effect, if any, should improve the performance of the later trials within each condition (i.e. the high difficulty trials) and hence reducing the behavioral difference between the low and high difficulty trials. However, even under a potential practice effect, robust differences in the behavioral measures were still observed between the low and high difficulty trials. On the other hand, as the order of the four conditions is counterbalanced across subjects, the low, medium and high difficulty trials of the four conditions were interleaved within the experiment which reduced the chance of having the effect of fatigue and boredom biasing the data of a particular difficulty level. Future studies with a larger sample size may investigate how EEG activities may be affected if the order of difficulty level is changed.

With the goal of bridging the gap between fundamental neuroscience research and real-world application (e.g. evaluating the real-time mental workload of students in a classroom setting), our study has provided a proof of concept in using single channel frontal EEG for short term mental workload detection. The EEG-based workload detection provides alternative approach to evaluate the study progress of students by monitoring the physiological response. The application can also be extended to self-study outside the classroom. When students are spelling vocabularies or solving an arithmetic problem, the short-term workload detection can provide feedback for self-evaluation. This study also shed new light on the possibility in developing a biomarker for quantifying mental workload and providing a real-time feedback on the dynamic change of mental workload.

## Supporting information

S1 Dataset EEG and behavioral data of 20 subjects. (ZIP)
